# Supplementary material for: Three putative DNA methyltransferases of Verticillium dahliae differentially contribute to DNA methylation that is dispensable for growth, development and virulence
Source: Epigenetics Chromatin. 2021 May 3;14:21. doi: 10.1186/s13072-021-00396-6 (PMC8091789; doi:10.1186/s13072-021-00396-6)
Supplement: Supplementary file 2 — Additional file 2: Figure S1. Phylogenetic tree of DNA methyltransferases. Figure S2. Verticillium dahliae ∆Dim5 loses H3K9me3. Figure S3. Growth assay of complementation strains. Figure S4. Stress assay pictures at 10 dpi. Figure S5. DNA methylation in CG context. Figure S6. DNA methylation in CHG context. Figure S7. DNA methylation in CHH context. Figure S8. DNA methylation over the genome. Figure S9. Occurrence of CG, CHG and CHH sites in methylated and non-methylated transposable elements. Figure S10. Comparison of protein domain structure of C. neoformans and V. dahliae Dnmt5. Figure S11. Distribution of H3K9me3 domain lengths. Figure S12. Genes induced in Hp1 and Dim5 mutants cluster more often than expected based on chance. Figure S13. Transposons induced in Hp1 and Dim5 mutants cluster more often than expected based on chance. Figure S14. Clusters of genes and transposons over all chromosomes. [file 13072_2021_396_MOESM2_ESM.docx]

# ADDITIONAL FIGURES


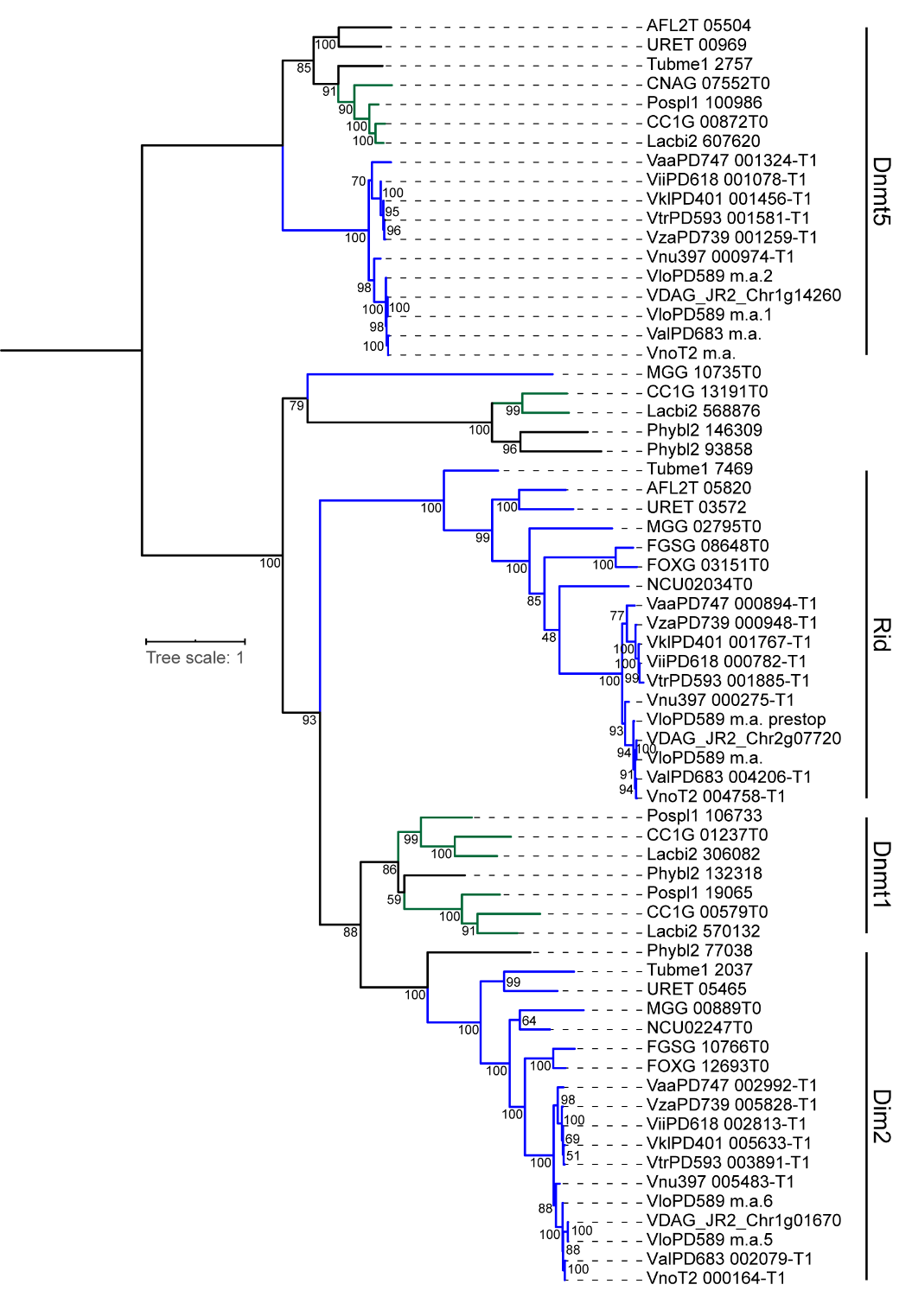


**Figure S1. Phylogenetic tree of DNA methyltransferases.** Gene codes containing “m.a.” were manually added, as they were missed in the predicted proteomes.

**
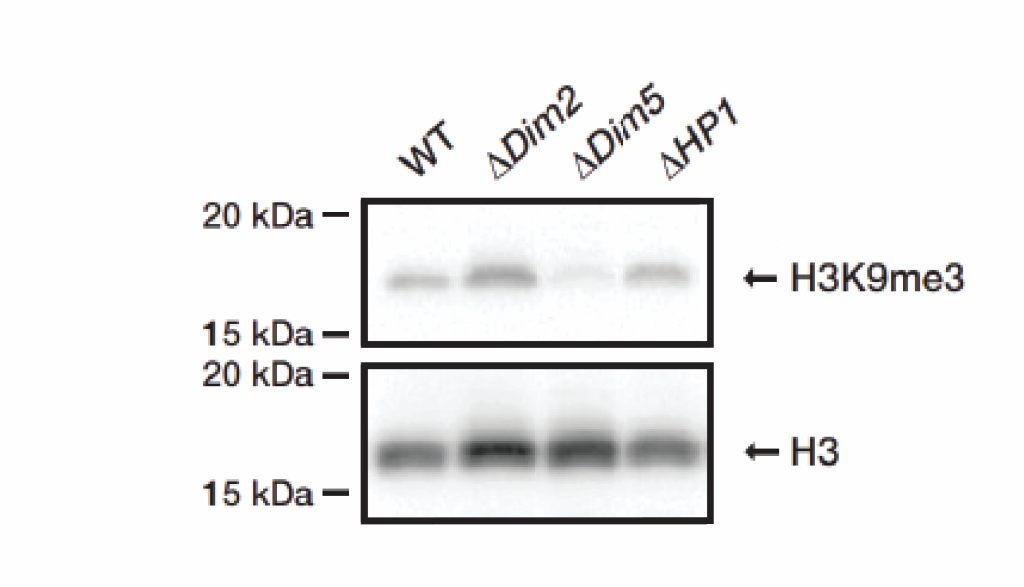
**

**Figure S2. Verticillium dahliae ∆Dim5 loses H3K9me3.** Western blot on nuclear protein extracts of V. dahliae wild-type and mutants show loss of H3K9me3 in the ∆Dim5 deletion mutant.

**
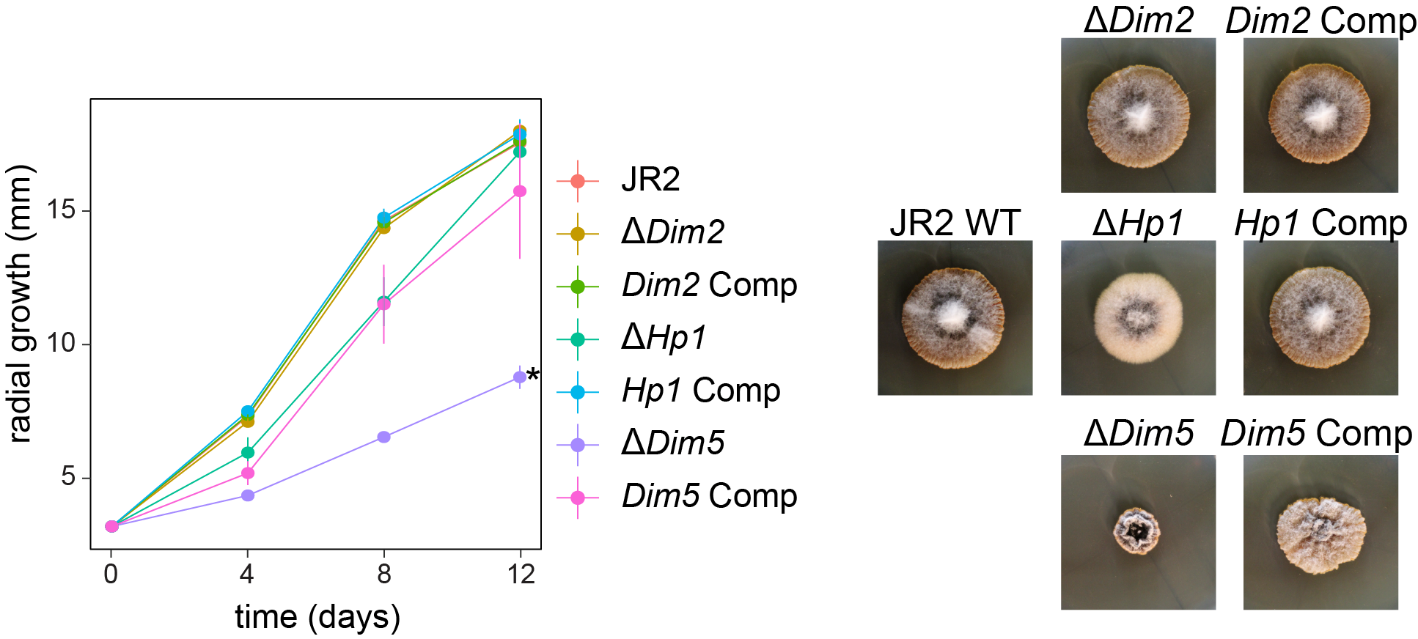
Figure S3. Growth assay of complementation strains.** Radial growth of wild-type, mutants and complementation strains over 12 days. Vertical lines represent the standard error of 8 measured colonies. Pictures showing representative colony morphology after 12 days of growth are shown on the right. Statistically significant differences from wild-type at 12 dpi (Wilcoxon Signed Rank, p < 0.01) are indicated with asterisks.

**
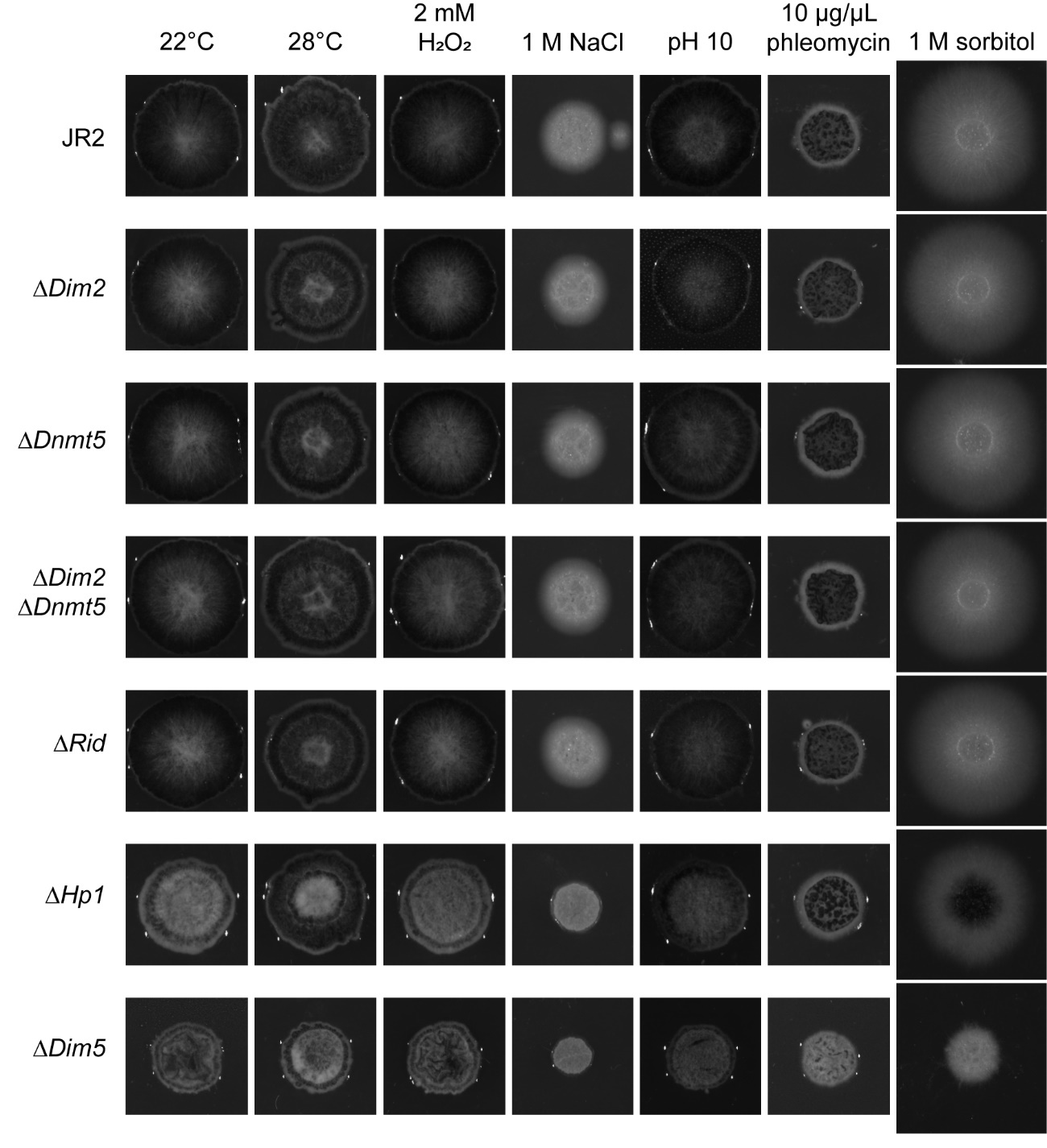
**

**Figure S4. Stress assay pictures at 10dpi.** Colony photographs as taken by the ChemiDoc MP imaging system underlying data of Figure 2D.


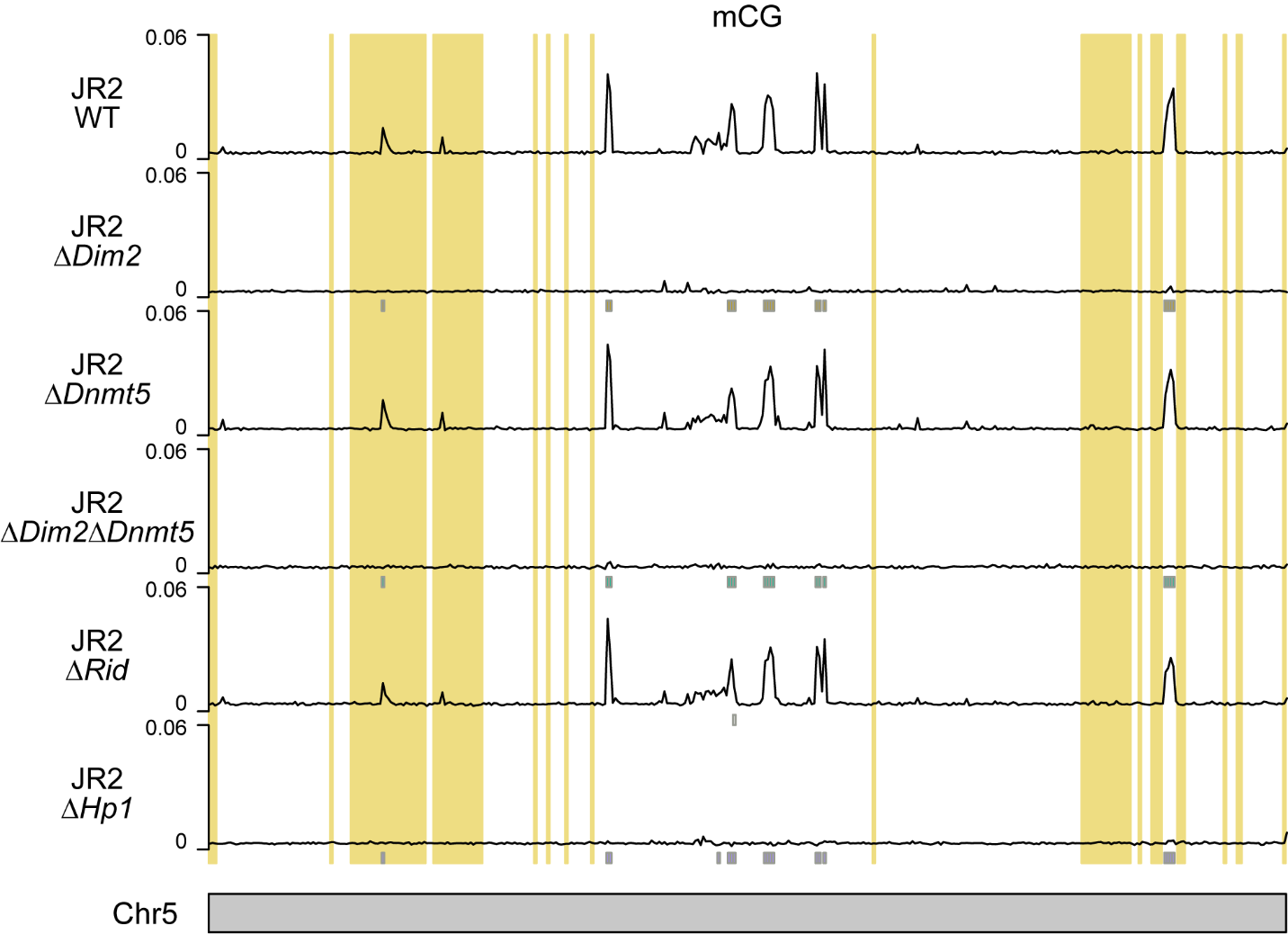


**Figure S5**. **DNA methylation in CG context.** Whole-chromosome plot displaying the fraction of methylated cytosines for non-overlapping 10 kb windows in CG context for WT, and DNA methyltransferase and Hp1 deletion mutants with chromosome 5 as an example. Grey boxes, displayed below the DNA methylation tracks, indicate the hypomethylated windows in CG context from Table 1. Previously defined LS regions (Cook et al., 2020) are highlighted in yellow.


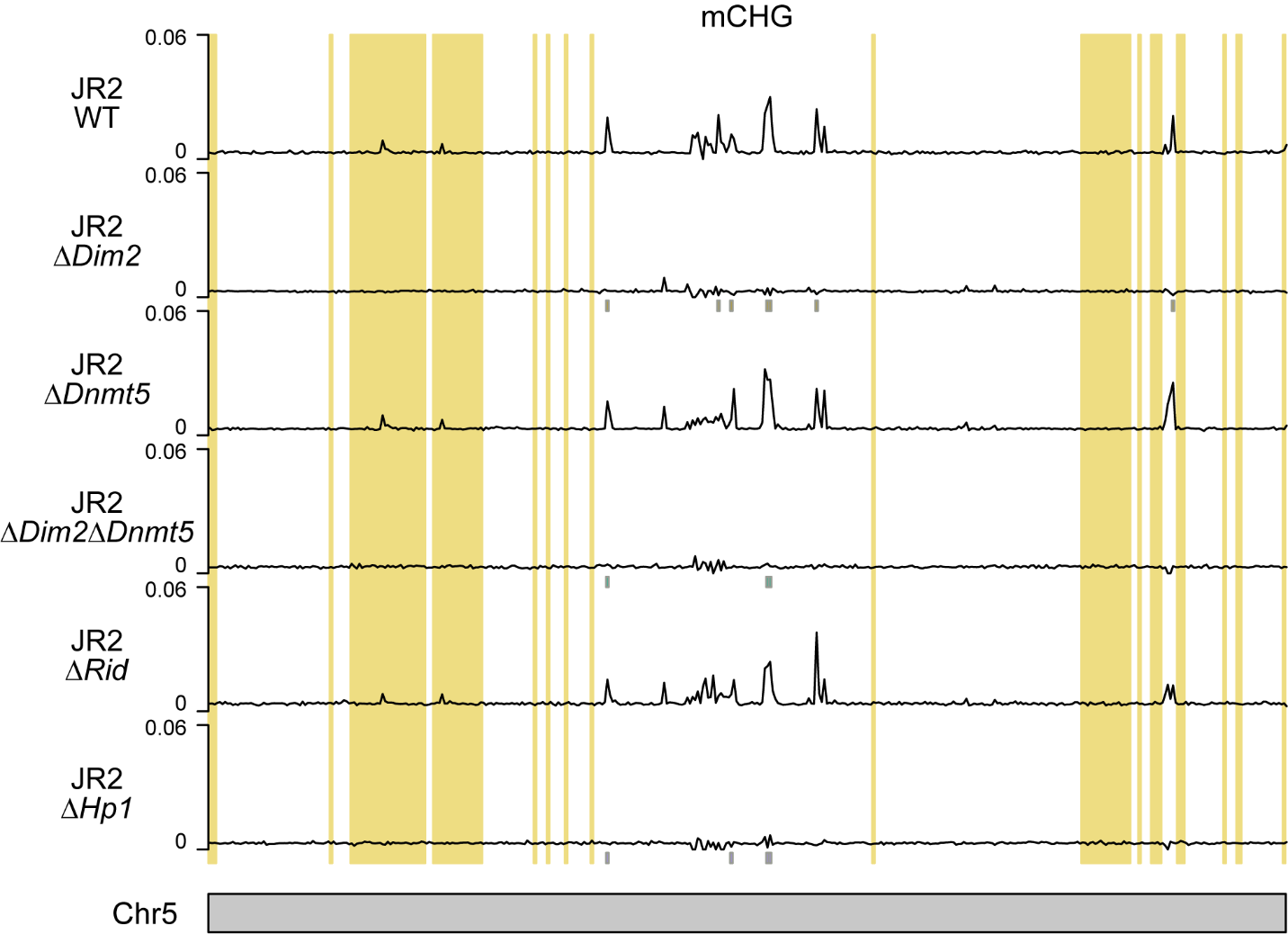


**Figure** **S6. DNA methylation in CHG context.** Whole-chromosome plot displaying the fraction of methylated cytosines for non-overlapping 10 kb windows in CHG context for WT, and DNA methyltransferase and Hp1 deletion mutants with chromosome 5 as an example. Grey boxes, displayed below the DNA methylation tracks, indicate the hypomethylated windows in CHG context from Table 1. Previously defined LS regions (Cook et al., 2020) are highlighted in yellow.


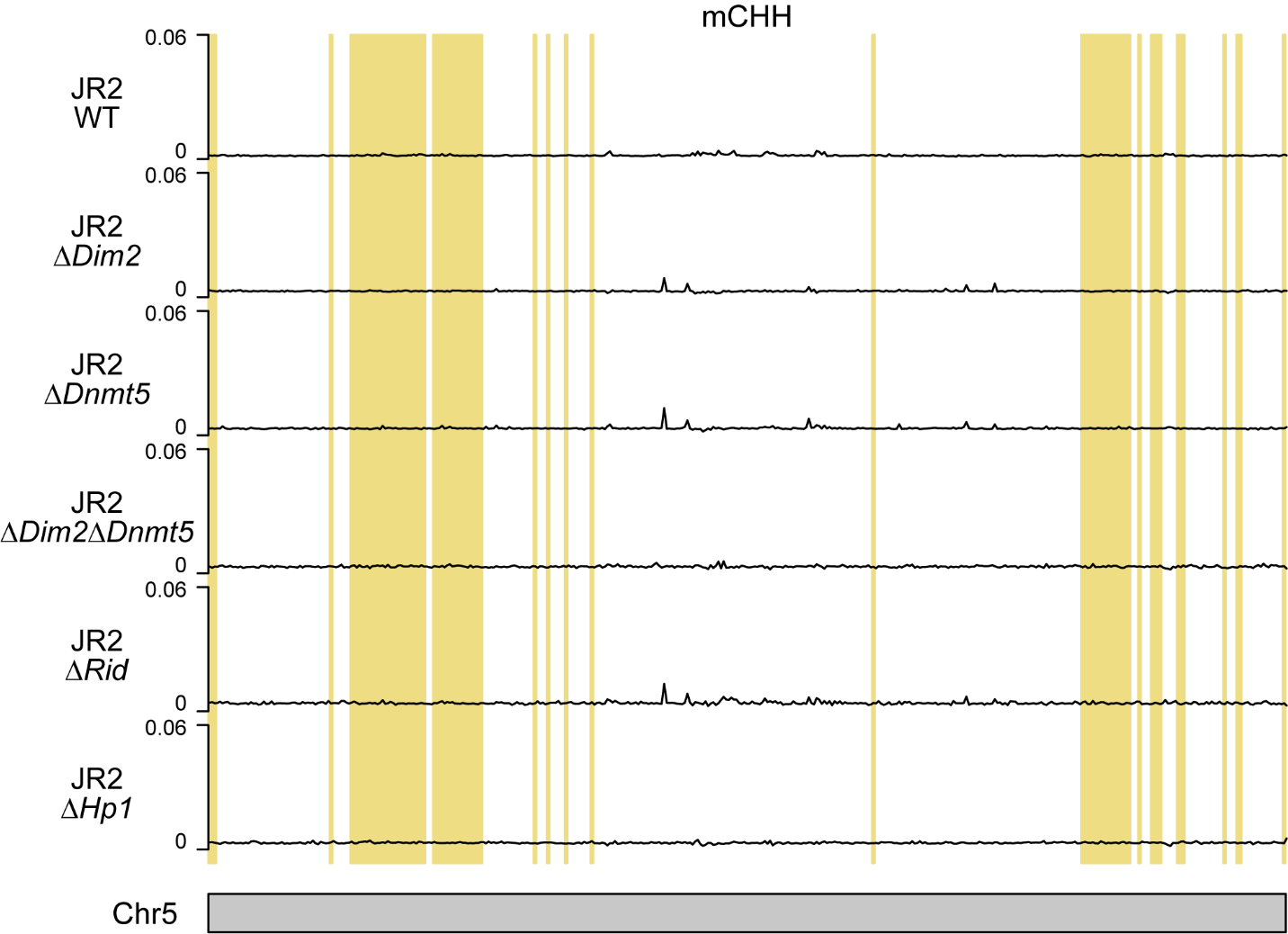


**Figure S7. DNA methylation in CHH context.** Whole-chromosome plot displaying the fraction of methylated cytosines for non-overlapping 10 kb windows in CHH context for WT, and DNA methyltransferase and Hp1 deletion mutants with chromosome 5 as an example. Colored boxes, displayed below the DNA methylation tracks, indicate the hypomethylated windows in CHH context from Table 1. Previously defined LS regions (Cook et al., 2020) are highlighted in yellow.


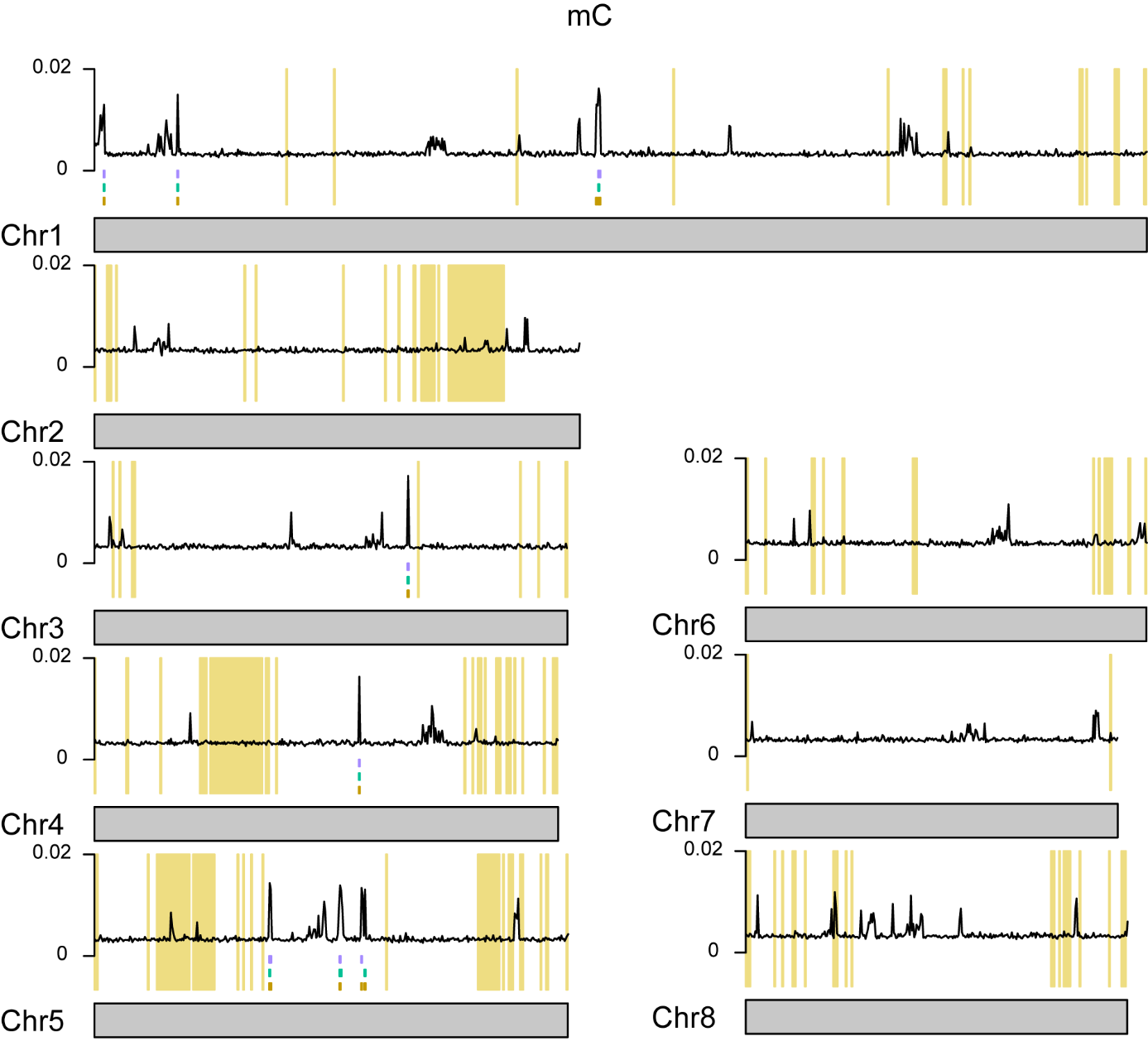


**Figure S8. DNA methylation over the genome.** Whole-chromosome plot displaying the fraction of methylated cytosines for non-overlapping 10 kb windows for wild-type over the whole genome. Colored boxes, displayed below the DNA methylation track indicate the hypomethylated windows from Table 1. Brown ΔDim2, green ΔDnmt5, teal ΔDim2-ΔDnmt5, blue ΔRid, purple ΔHp1. Previously defined LS regions (Cook et al., 2020) are highlighted in yellow.


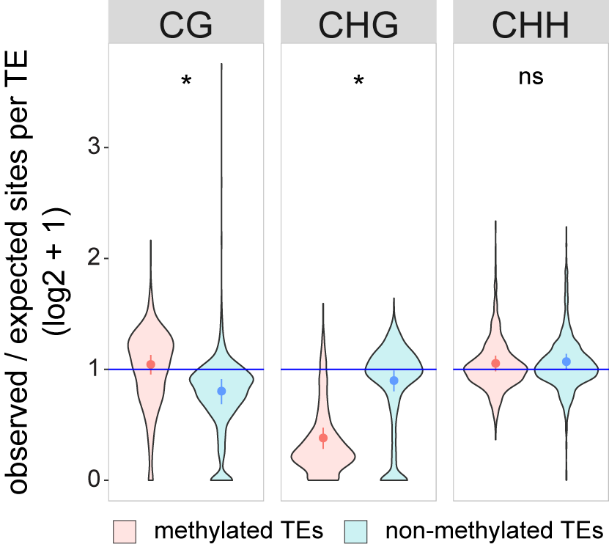


**Figure S9. Occurrence of CG, CHG and CHH sites in methylated and non-methylated transposable elements.** The observed sites per TE were compared to the number of expected sites based on sequence composition per TE.


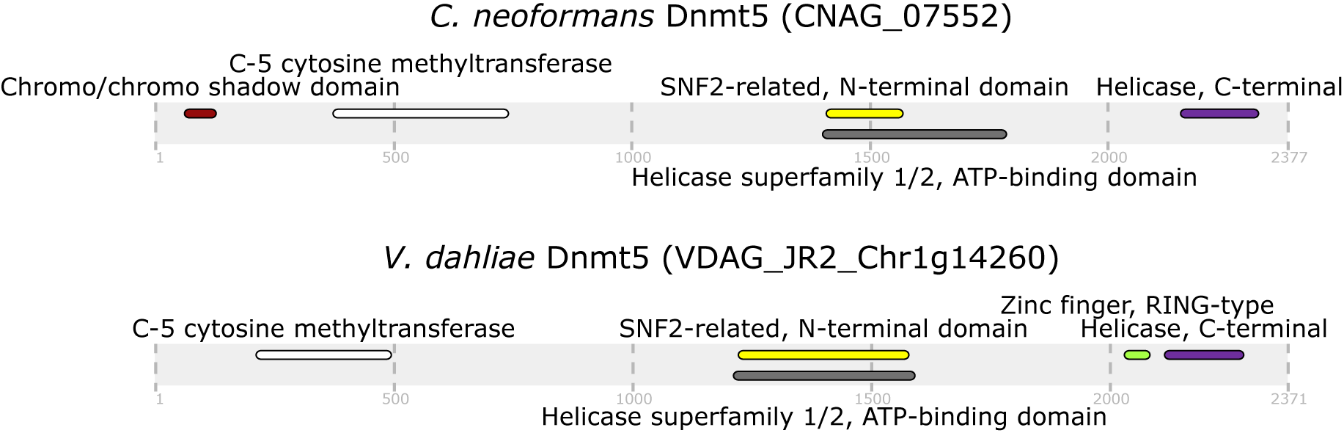


**Figure S10.** Comparison of protein domain structure of C. neoformans and V. dahliae Dnmt5.


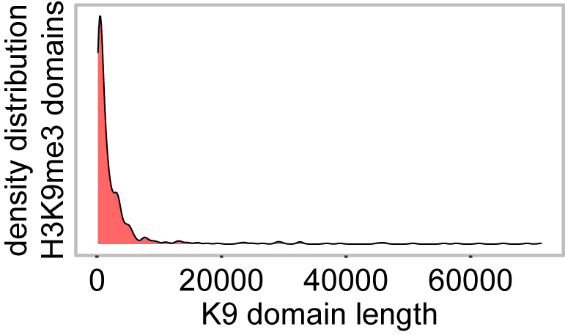


**Figure S11. Distribution of H3K9me3 domain lengths.**


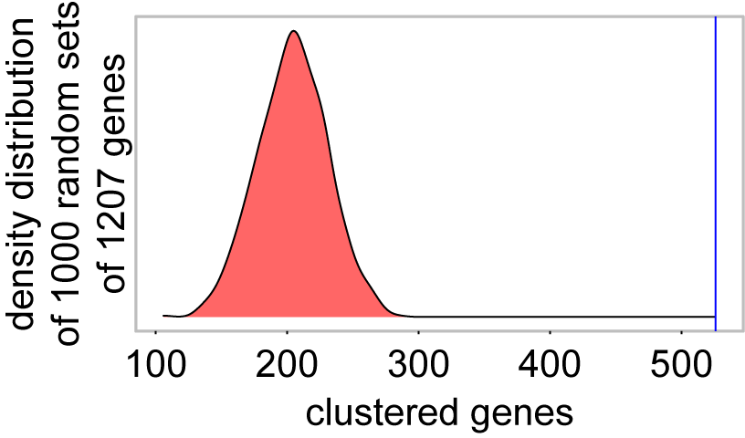


**Figure S12. Genes induced in** **Hp1 and Dim5 mutants cluster more often than expected based on chance.** The 526 out of 1207 induced genes (blue vertical line) that cluster in the genome are more than would be expected based on 1000 random sets of 1207 genes.


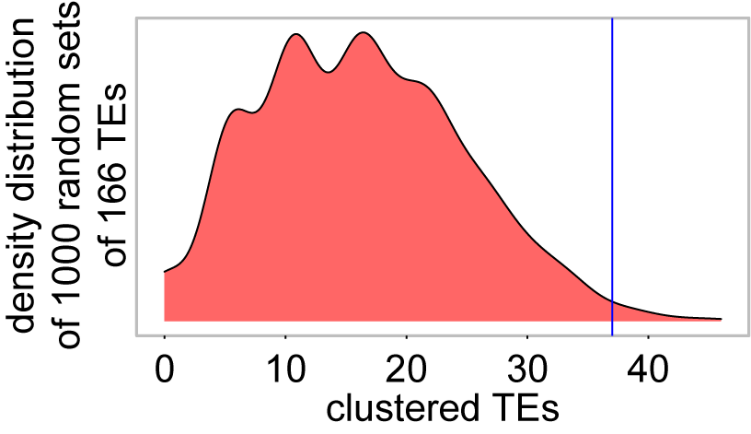


**Figure S13. Transposons induced in Hp1 and Dim5 mutants cluster more often than expected based on chance.** The 37 out of 166 induced TEs (blue vertical line) that cluster in the genome are more than would be expected based on 1000 random sets of 166 TEs.


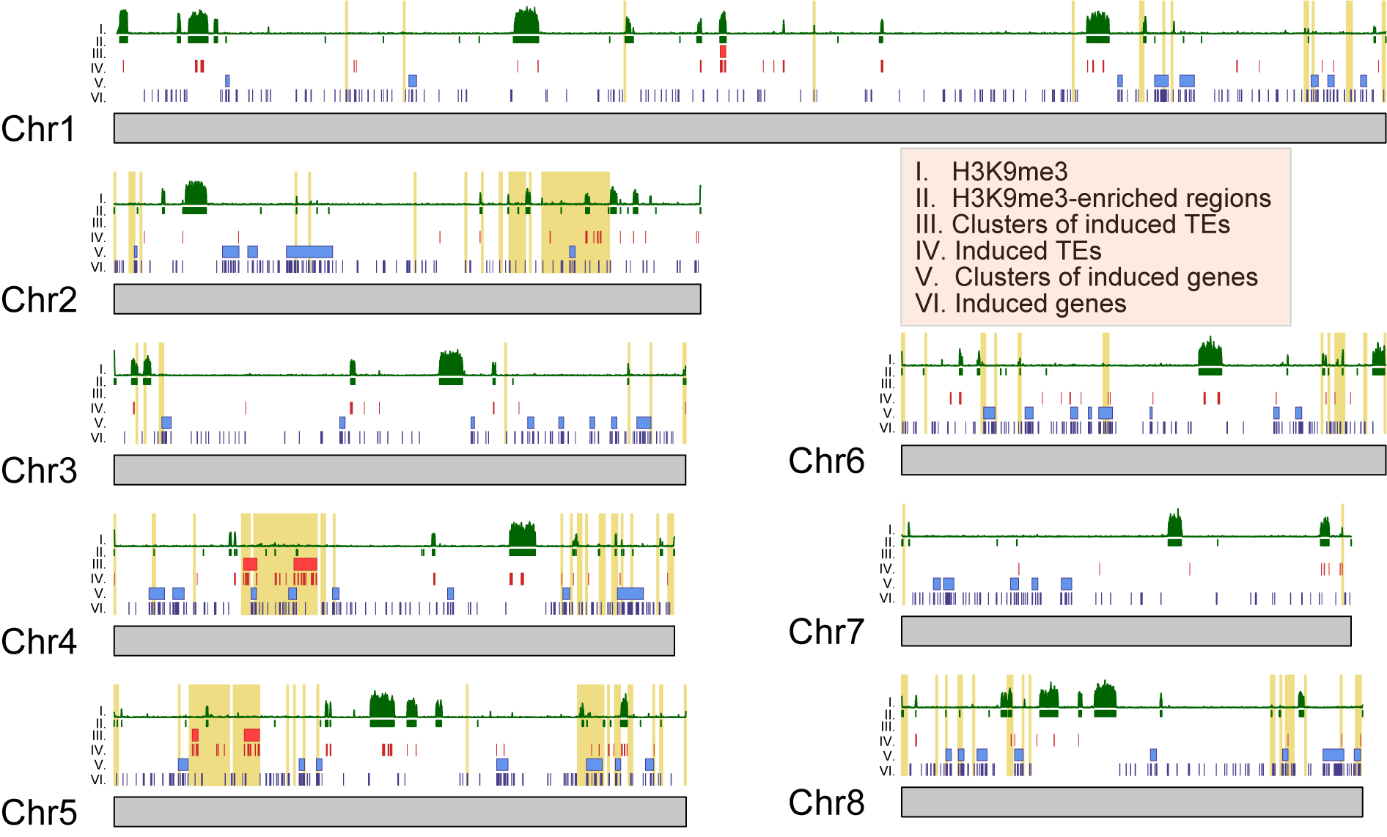


**Figure S14. Clusters of genes and transposons over all chromosomes** Whole-chromosome plots displaying the location of induced genes (in blue) and transposons (in red). Clusters of induced genes and transposons are indicated as blue and red rectangles, respectively. H3K9me3-ChIP signal along the chromosomes is indicated in green in the upper track. Previously defined LS regions (Cook et al., 2020) are highlighted in yellow.
